# Characterization of Steel Slag Filler and Its Effect on Aging Resistance of Asphalt Mastic with Various Aging Methods

**DOI:** 10.3390/ma14040869

**Published:** 2021-02-11

**Authors:** Minghua Wei, Shaopeng Wu, Haiqin Xu, Hechuan Li, Chao Yang

**Affiliations:** 1 School of Transportation, Wuhan University of Technology, Wuhan 430070, China; weiminghua@whut.edu.cn; 2 State Key Laboratory of Silicate Materials for Architectures, Wuhan University of Technology, Wuhan 430070, China; hbyangc@whut.edu.cn; 3 Faculty of Engineering, China University of Geosciences, Wuhan 430074, China; lihechuan@cug.edu.cn

**Keywords:** steel slag filler, characterization, asphalt mastic, aging methods, resistance

## Abstract

Steel slag is the by-product of the steelmaking industry, the negative influences of which prompt more investigation into the recycling methods of steel slag. The purpose of this study is to characterize steel slag filler and study its feasibility of replacing limestone filler in asphalt concrete by evaluating the resistance of asphalt mastic under various aging methods. Firstly, steel slag filler, limestone filler, virgin asphalt, steel slag filler asphalt mastic and limestone filler asphalt mastic were prepared. Subsequently, particle size distribution, surface characterization and pore characterization of the fillers were evaluated. Finally, rheological property, self-healing property and chemical functional groups of the asphalt mastics with various aging methods were tested via dynamic shear rheometer and Fourier transform infrared spectrometer. The results show that there are similar particle size distributions, however, different surface characterization and pore characterization in the fillers. The analysis to asphalt mastics demonstrates how the addition of steel slag filler contributes to the resistance of asphalt mastic under the environment of acid and alkaline but is harmful under UV radiation especially. In addition, the pore structure in steel slag filler should be a potential explanation for the changing resistance of the asphalt mastics. In conclusion, steel slag filler is suggested to replace limestone filler under the environment of acid and alkaline, and environmental factor should be taken into consideration when steel slag filler is applied to replace natural fillers in asphalt mastic.

## 1. Introduction

On account of the outstanding performances of asphalt mixtures, asphalt pavements have been widely used in road construction. Generally, asphalt pavements are considered as a system of coarse aggregates, fine aggregates and asphalt mastic in previous research [1,2]. In the system, the addition of fillers to bitumen forms the asphalt mastic, which fills the interstices between aggregates and enhances the bonding performance [3]. Former research has demonstrated that it is the properties of asphalt mastics that influence the resistance to high-temperature deformation and low-temperature cracking of asphalt mixtures [4,5]. Due to the multiple functions of the fillers in asphalt mastic, the qualities of fillers have a significant impact on the asphalt mixture [6]. Nevertheless, the advancement of road construction projects in China, especially on the highway, has consumed consequential high-quality mineral fillers, which requires the novel substitution of analogous properties to replace natural mineral fillers urgently [7].

Steel slag, the main by-product in the steel-making industry, accounts for over 10% of crude steel production in the world [8,9]. However, the negative impact of steel slag on the environment, including occupation of the field, secondary pollution to water and soil and potential pollution of the heavy metal, demands the rescue and management of steel slag as soon as possible [10,11]. Fortunately, the properties of high abrasion resistance, tough surface characteristics, strong alkalinity and outstanding mechanical properties contribute to the application of steel slag as aggregates in asphalt concrete [12,13,14]. In comparison to natural aggregates, such as basalt, limestone and diabase, the high abrasion resistance and the tough surface characteristics of steel slag increase the skid resistance of asphalt pavement sharply [15]. Meanwhile, the strong alkalinity of steel slag enhances the “bond” in the aggregate–asphalt system, which improves the anti-water damage performances of asphalt concrete [16,17,18]. Moreover, recycling of steel slag as aggregate in asphalt pavement can subside the continuous demand of natural aggregates availably, limit the negative influence of steel slag on environment and even decrease the cost and energy consumption of road construction in certain degree [19]. These advantages make steel slag a superior substitute for natural aggregates.

Nonetheless, previous research on the recycling of steel slag in asphalt concrete attracts concern for, mainly, the properties of asphalt pavement with steel slag replacing coarse aggregates and fine aggregates; research on the properties of asphalt pavement with steel slag fillers(SSF) replacing the fillers is scarce. At present, the research focuses on the properties of asphalt mastics with SSF replacing natural mineral fillers in a regular environment. Li et al. [20] discussed the effect of steel slag filler milled from raw steel slag with different particle sizes on rheological properties of asphalt mortar and claimed that steel slag fillers can be used as potential materials to replace limestone fillers. Tao et al. [21] came to a similar conclusion and proposed that the steel slag fillers content should be lower than 75% by volume. Kong et al. [22] selected different basic oxygen furnace slag filler to investigate its influence on properties of asphalt mastic and the results showed the fillers could increase high temperature anti-rutting stability of asphalt concrete but reduce low-temperature flow performance of asphalt mastic. Notwithstanding, the performance of asphalt mastics with SSF stand a good chance of differing from that with limestone fillers (LF) under different aging methods, due to the fact that the pore-structure of steel slag has the potential to reflect less energy and refract more energy more easily [23]. In addition, the reaction between different environment and sufficient f-CaO in SSF can cause the confusion of reacted order, such as in acid, alkali, NaCl and pure water environment, which correspond to the area of acid rain, salt lick and normalization [24,25,26]. Meanwhile, the environment of heat, oxygen and ultraviolet also should be taken into the consideration [27]. Therefore, it is significant to figure out the environmental influence on the asphalt mastics with SSF and the difference with the asphalt mastics with LF.

This research aims to study the feasibility of SSF replacing LF in asphalt concrete by evaluating the resistance of asphalt mastic under various aging methods. Firstly, the particle size distribution, surface characterization and pore characterization of SSF and LF were evaluated. Virgin asphalt (VA), steel slag filler asphalt mastic (SSFA) and limestone filler asphalt mastic (LFA) were prepared. Then, solution aging (acid solution/alkali solution/NaCl solution/pure water), thin-film oven test (TFOT) aging, UV aging and TFOT+UV aging were applied to the asphalt mastics. The rheological property, self-healing property and chemical functional groups of SSFA and LFA with various aging methods were tested, respectively.

## 2. Materials and Specimens Preparation

### 2.1. Raw Materials

In this research, the commonly used bitumen (AH-70 asphalt), provided from Guochuang Co., Ltd.,Wuhan, Hubei, China, was used as binder in asphalt mastic. Steel slags with a particle size of 2.36–4.75 mm, supplied from Baotou Iron and Steel (Group) Co. Ltd., Baotou, Inner Mongolia, China, were selected to prepare steel slag fillers (SSF). The basic properties of AH-70 asphalt and steel slag can be seen in Table 1.

Steel slags were crushed through the ball mill for 25 min and sieved into fillers with a particle size less than 0.075 mm. As the reference fillers for comparison with SSF, limestone fillers (LF) were purchased from Maliang Aggregates, Jingmen, China. The basic properties of the fillers are shown in Table 2 [29].

### 2.2. Preparation of Asphalt Mastic

In this study, AH-70 asphalt, SSF and LF were chosen to prepare asphalt mastic. The consumption of asphalt and fillers were determined by a qualified mix proportion of asphalt concrete meanwhile asphalt aggregate ratio was 4.9%. Therefore, the volume ratio of fillers to asphalt was determined as 0.3.

Preparation steps of asphalt mastic were as follows:

1. The asphalt and fillers were heated to target temperatures in the oven (AH-70 asphalt of 120 °C and fillers of 150 °C);
2. Then, asphalt was removed to the containers kept in an oil bath to hold the temperature. Asphalt was pre-mixed with rotating speed limited at 500 rpm for 5 min;
3. Fillers were added into a container gradually with consistent rotating speed. Until all the fillers were added, the speed was increased to 1500 rpm for 10 min;
4. Finally, asphalt mastic was transferred to other containers for storage.
5. Eventually, virgin asphalt (VA), steel slag fillers asphalt mastic (SSFA) and limestone fillers asphalt mastic (LFA) were prepared.

## 3. Research Methods

### 3.1. Material Characteristics of Fillers

The particle size distribution of the fillers was evaluated by laser particle size analyzer, Mastersizer 2000, Malvern panalytical, Malvern, UK. A scanning electron microscope, QUANTA FEG 450, made by FEI, Hillsboro, OR, USA, was used to characterize the surface characteristics of the two fillers. To obtain the characteristics of the pores in the fillers, samples were measured by ASAP 2020M manufactured by Micromeritics, Norcross, GA, USA.

### 3.2. Aging Methods

#### 3.2.1. TFOT Short-Term Aging

In accordance with the “Standard Test of Bitumen and Bituminous Mixtures for Highway Engineering” (JTG E20-2011) [28], the samples after short-term thermal aging were obtained by the thin-film oven test (TFOT). The conditions of standardization were (163 ± 1) °C for 5 h.

#### 3.2.2. Ultraviolet Aging

Ultraviolet (UV) aging was conducted after the short-term aging, the asphalt mortars were placed into the oven at 50 °C for UV aging, within a period of 3 days. The UV radiation was reached to 50 w/m^2^.

#### 3.2.3. Solution Aging

Acid solution (H2SO4/HNO3/water solution, pH = 3), alkali solution (NaOH solution, pH = 11), 10 wt.% NaCl solution (Sinopharm Chemical Reagent co.,Ltd, Shanghai, China) and pure water were used to perform solution aging. Asphalt mastics of 6 g were taken out to the glass dish with the radius of 49 mm and the height of 18 mm, then kept in the oven at 150 °C for half an hour to cover the bottom of the dish. Then, the samples of asphalt mastic with the thickness of about 0.76 mm were prepared. Solutions of 40 mL were poured into the dish to submerge the asphalt mastics, and the solutions were renewed every two days to keep the stability of pH value. After the aging of ten days, the samples were cleaned by pure water and kept in the oven of 150 °C for half an hour to remain dry. SSFA and LFA aged by acid solution, alkali solution, NaCl solution and pure water were named, respectively, SSFA-1, SSFA-2, SSFA-3 and SSFA-4; LFA-1, LFA-2, LFA-3 and LFA-4.

### 3.3. Rheological Properties Test

In this research, the rheological property of the asphalt mastics was tested by Dynamic Shear Rheometer (DSR), MCR101, Anton Paar, Graz, Austria. Temperature sweep was performed firstly in the range of 30 °C to 80 °C fixed at 10 rad/s. Then, the frequency sweep was performed at the same temperature range to temperature sweep. Twelve temperature levels were obtained at temperature interval of 5 °C. After reaching target temperature, the samples were scanned in therange of 0.1~100 Hz of frequency.

### 3.4. Chemical Structure Test

The chemical structures of VA and extractive asphalt mastics before and after the agings were evaluated by a Fourier transform infrared spectrometer (FTIR, Nexus, ThermoNicolet Crop., Waltham, MA, USA). In this paper, FTIR tests were performed under a scanned range between 4000 and 400 cm^−1^ and the scanning resolution was 4 cm^−1^.

### 3.5. Experimental Plan

**Figure 1:**
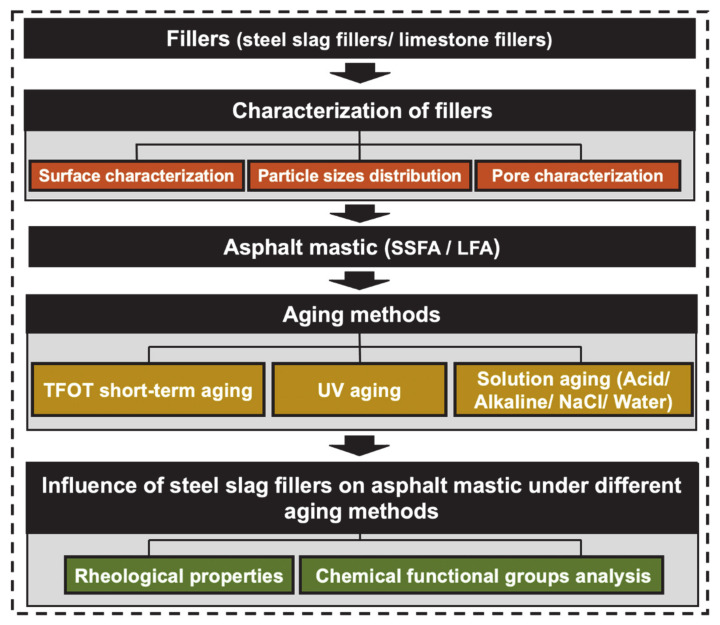
The flow chart of this research.

The experimental plan is shown in Figure 1.

## 4. Results and Discussion

### 4.1. Characterization of Fillers

#### 4.1.1. Particle Size Distribution Analysis

**Figure 2:**
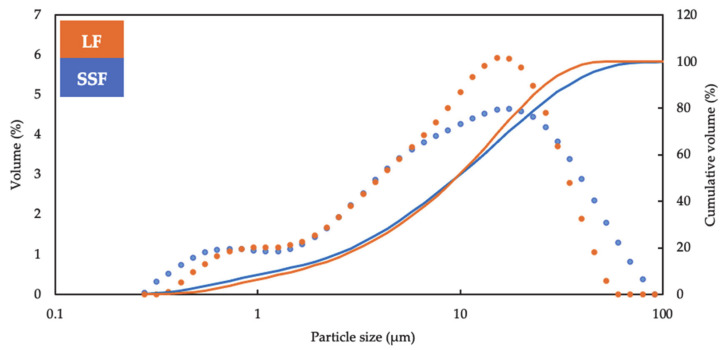
Particle size distribution of SSF and LF.

Particle size distribution analysis of the fillers (SSF and LF) are shown in Figure 2. The particle size distribution of LF is at the range of around 0.3 µm to 60 µm and that of SSF is at the range of around 0.3 µm to 100 µm. The particle size of SSF is more concentrated at the range of around 0.3 µm to 0.8 µm and 30 µm to 100 µm while particle size of LF is more concentrated at the range of around 6 µm to 30 µm. At the residual range (round 0.8 µm to 6 µm), the distribution of the fillers is similar significantly. In general, the particle size distribution of SSF and LF presents similitude, especially at the range from 0.3 µm to 10 µm). However, the distinction in the particle sizes distribution indicates SSF possessed more uniformed distribution than LF. The uniformity might be caused by the method of grinding by manual in laboratory. Moreover, the resemblance in particle size distribution of the fillers contributes to the replacement of SSF to LF as fillers in asphalt concentrate from the perspective of gradation.

#### 4.1.2. Surface Characterization

**Figure 3:**
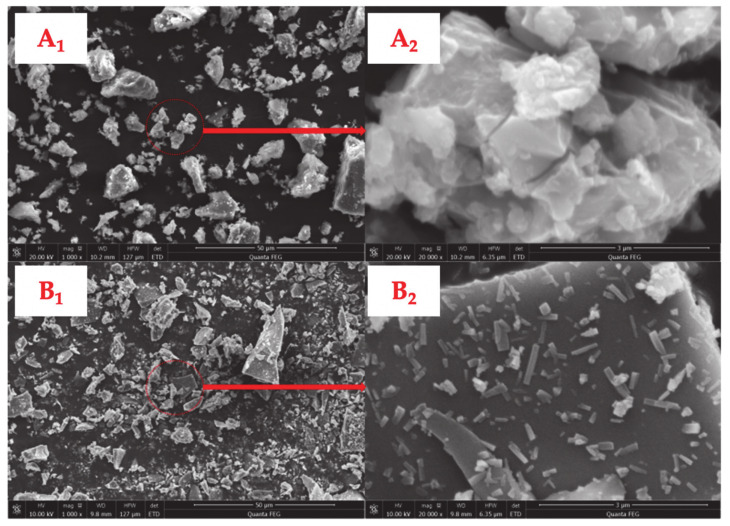
Surface characterization of fillers: SSF (**A_1_**,**A_2_**) and LF (**B_1_**,**B_2_**).

The surface characterizations of SSF and LF tested through SEM method are shown in Figure 3. The surface of the SSF is observed to be rugged, which leads to an increase in cohesion between asphalt binder and fillers. Conversely, the surface of LF is markedly smoother than that of SSF, in which the former is less irregular-shaped, rough, and bumpy. These characterizations might have a negative impact on effective cohesion with asphalt binder and accordingly lead to poorer strength and water resistance. The raw materials of the fillers could account for their totally different surface characterization. SSF is obtained from steel slag whose surface remained rough and adhered numerous tiny particles though steel slag is grounded into filler, while LF is obtained by crushing limestone. Analogous to the application of steel slag in asphalt concrete, the unique surface characterizations of SSF could cause an increased consumption in the amount of asphalt required when applied in asphalt concrete. However, the usage of SSF is not supposed to increase the practical cost due to the fact that steel slag is actually considered as solid waste with cheapness.

#### 4.1.3. Pore Characterization

**Figure 4:**
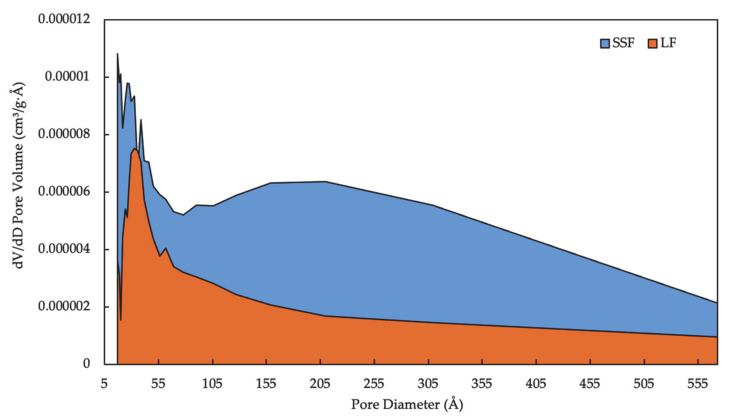
Pores distribution of SSF and LF.

The quantification of pore characterizations about SSF and LF is shown in Table 3. The surface area could present the number of pores in the fillers, and the BET model that was accepted most widely is selected to calculate the surface area. Compared to LF, there is nearly 100% increment of BET surface area in SSF. Similarly, the single point adsorption total volume of pores in SSF is more than that in LF—0.006174 cm^3^/g as opposed to 0.002228 cm^3^/g. Furthermore, less obvious changes are recorded in the BJH desorption average pore diameter of the fillers, where the values remain relatively unchanged orders of magnitude but differed greatly. The values have also confirmed the appearance shown in Figure 3. Figure 4 describes the pore distribution of SSF and LF. The dV/dD pore volume in SSF fluctuates violently between 17 Å and 100 Å, during which the maximum had risen above 0.00001 cm^3^/g·Å. The trends for the dV/dD pore volume in LF are almost the same, though their figures have obvious difference, with the former always standing larger than the latter. The closed area of the curves demonstrates the total pore volume of the fillers, which indicates more possession of pores in SSF. Furthermore, BET is the acronym of S. Brunauer, P. Emmett and E. Teller who proposed multi-molecular layer adsorption theory; BJH is a model named after the names of Barret, Joyner, and Halenda.

### 4.2. Influence of Steel Slag Fillers on Asphalt Mastic of Rheological Properties

**Figure 5:**
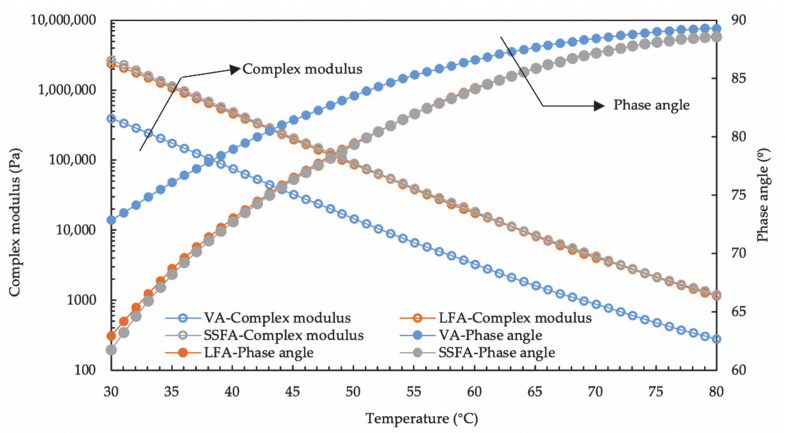
Temperature sweep analysis of VA, SSFA and LFA.

**Figure 6:**
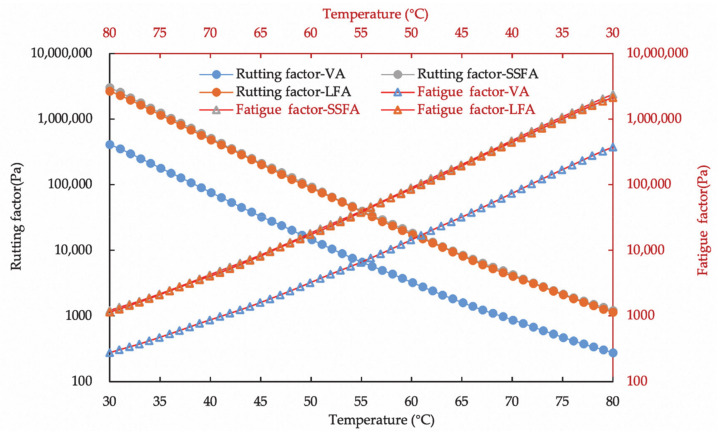
Rutting factor and fatigue factor of VA, SSFA and LFA.

Figure 5 illustrates the results of temperature sweep analysis of VA, SSFA and LFA. It can be inferred that complex modulus of VA is the least while phase angle of VA is the most, which means VA has the minimum stiffness and most viscous components. Meanwhile, the similarity of the complex modulus and phase angle of SSF and LF is significantly obvious though there is still a slight difference. SSF possesses a larger complex modulus but lower phase angle. Compared to VA, a higher complex modulus of both SSF and LF indicates that the application of fillers heightened the resistance of the asphalt mastic to high-temperature deformation. Moreover, the slightly discrepancy of SSF and LF in rheological properties might be caused by the different characterization of fillers, especially the pore characterization and the raw materials. The characteristic with more pores of SSF has the potential to cause preferable absorption and combination with asphalt, which contributes to the increment in the complex modulus. In addition, the raw material of SSF is in stark contrast with that of LF: steel slag is rigid abundantly as far as the hardness is concerned. The rutting resistance of asphalt mortar is quantified by the rutting factor, which is expressed as G*/sinδ and related to the high-performance resistance of asphalt concrete. The fatigue factor, expressed as G*sinδ, the speed of energy loss under repeated loading. The fatigue damage and lifetime of an asphalt mixture have a proportional relationship with the energy loss during a cyclic loading process [30]. As shown in Figure 6, the rutting factor of SSFA and LFA is larger than that of VA, meanwhile the trends for rutting factor and fatigue factor of the samples are almost the same. It demonstrates that all samples containing fillers own better rutting resistance than that of VA. Furthermore, SSFA possesses similar but slightly superior resistances than that of LFA, which is conducive for SSF to the replacement of the fillers in asphalt concrete. This should be considered as a result of the chemical effect by alkaline compositions in SSF and asphaltic acid in asphalt. The stiffness of SSF also contributes to a more stable structure in the asphalt mastic and moreover better resistance.

### 4.3. Influence of Steel Slag Fillers on Asphalt Mastic under Different Aging Methods and Its Mechanism

#### 4.3.1. Rheological Properties

**Figure 7:**
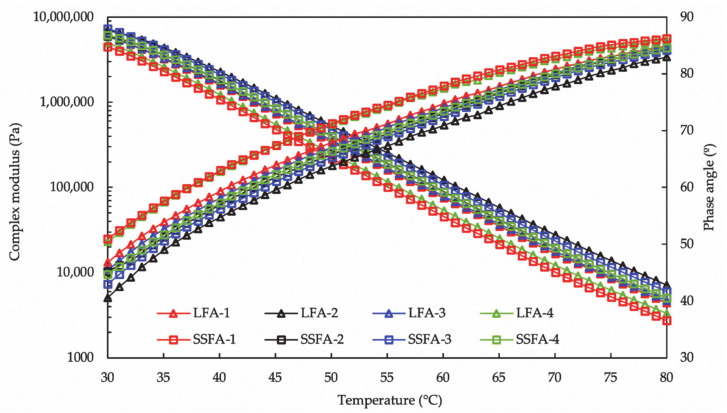
Temperature sweep analysis of SSFA and LFA after solution aging.

The complex modulus and phase angles of SSFA and LFA conditioned by different solutions to simulate the impact by four kinds of environment for ten days are shown in Figure 7. The differentia in the rheological properties can reflect the ageing states of the bitumen. During the aging process, the rheological properties of SSFA and LFA change obviously; the complex modulus of asphalt mastic increases, meanwhile the phase angles decreases. The order of the complex modulus of SSFA is SSFA-3 > SSFA-2 > SSFA-4 > SSFA-1, and that of LFA is LFA-2 > LFA-3 > LFA-1 > LFA-4. The complex modulus values of SSFA after the aging of NaCl solution and pure water are higher than that of LFA, while the situation is reversed under acid solution and alkaline solution. In addition, the order of the changing trend of the phase angle is consistent with the complex modulus. After the aging of NaCl solution and pure water, rather than the aging of acid solution and alkaline solution, the phase angles of SSFA are smaller compared to LFA. The results show that, as compared to LFA, the negative impacts of NaCl solution and pure water on SSFA about the rheological properties are more serious than acid solution and alkaline solution.

The complex modulus and the phase angle of the asphalt mastic after the aging process to that of the asphalt mastic before the aging process are applied to characterize the complex modulus and the phase angle changes during the aging process. According to Equations (1) and (2), the two rheological aging indexes are calculated.

(1) $$\mathrm{Complex}\;\mathrm{modulus}\;\mathrm{aging}\;\mathrm{index}=\frac{\mathrm{Aged}\;\mathrm{complex}\;\mathrm{modulus}}{\mathrm{Unaged}\;\mathrm{complex}\;\mathrm{modulus}}\times 100\;\%$$

(2) $$\mathrm{Phase}\;\mathrm{angle}\;\mathrm{aging}\;\mathrm{index}=\frac{\mathrm{Aged}\;\mathrm{phase}\;\mathrm{angle}}{\mathrm{Unaged}\;\mathrm{phase}\;\mathrm{angle}}\times 100\;\%$$

**Figure 8:**
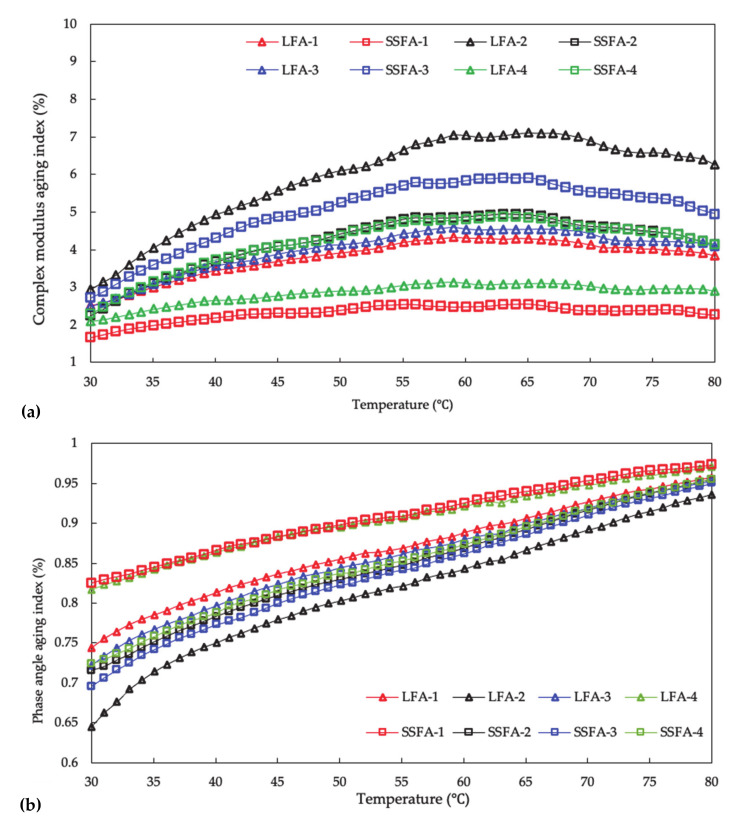
The changes of rheological properties of SSFA and LFA before and after solution aging. (**a**) Complex modulus aging index; (**b**) phase angle aging index.

The results in the rheological aging indexes of SSFA and LFA before and after aging are shown in Figure 8. With the increment of temperature, the complex modulus aging index shows a gradual upward trend from 30 °C, reaches a temporary peak at the range of 60 °C to 70 °C, and then gradually decreases. This might be due to the fact that a temperature barrier existed at the range of 60 °C to 70 °C, and the index would approach 1 once the barrier is broken. As for the phase angle aging index, it presents a similar trend of increasing and a different trend of approaching and concentrating.

From Figure 8, after the same conditioning time, the order of the complex modulus aging index of SSFA is SSFA-3 > SSFA-2 > SSFA-4 > SSFA-1; the order of the complex modulus aging index of LFA is LFA-2 > LFA-3 > LFA-1 > LFA-4. These tendencies are the same with the complex modulus.

The orders indicate the negative impact to the asphalt mastics of different solutions: NaCl solution > alkaline solution > pure water > acid solution (for SSFA); alkaline solution > NaCl solution > acid solution > pure water (for LFA). Moreover, the trend for the phase angle aging index corresponding to the negative impact are almost the same. Compared with LFA, SSFA presents a smaller complex modulus aging index and a larger phase angle aging index under the aging of acid solution and alkaline solution, whereas the opposite under the aging of NaCl solution and pure water. In summary, SSFA has a more outstanding performance in the environment of acid solution and alkaline solution but poor performance in the environment of NaCl solution and pure water than that of LFA.

**Figure 9:**
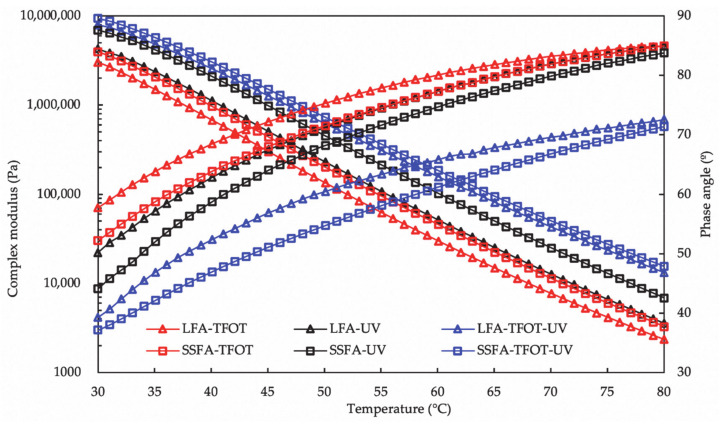
Temperature sweep analysis of SSFA and LFA after TFOT/UV/TFOT+UV aging.

The complex modulus and phase angles of SSFA and LFA conditioned by TFOT/UV/TFOT+UV aging are shown in Figure 9. The results clearly demonstrate the diversity of the effect of the asphalt mastics. Under the three aging methods, the complex modulus of SSFA is all larger than that of LFA, meanwhile the order of the changing range of the phase angle is consistent with the complex modulus. UV aging methods witness the greatest deviation of the complex modulus, which indicates that the resistance to UV radiation of the asphalt mastics differed significantly. Moreover, the resistance to TFOT aging is posterior, and that of TFOT+UV aging is the least.

**Figure 10:**
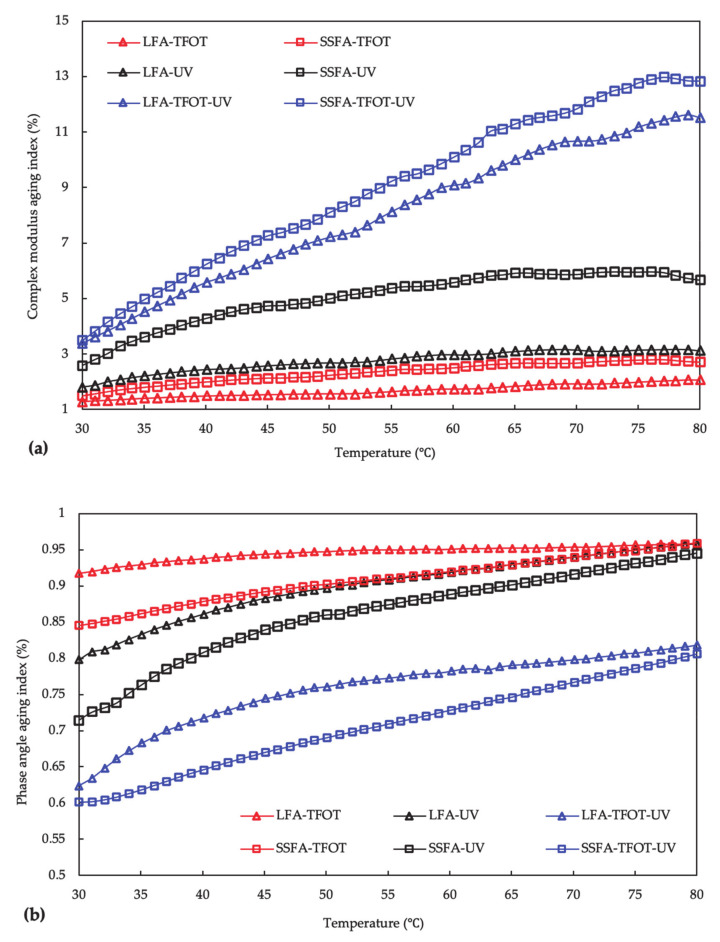
The changes of rheological properties of SSFA and LFA before and after TFOT/UV/TFOT+UV aging: (**a**) complex modulus aging index; (**b**) phase angle aging index.

The changes of rheological properties of SSFA and LFA before and after TFOT/UV/TFOT+UV aging are shown in Figure 10. Similar to the results in Figure 8, with the increment of temperature, the complex modulus aging index shows a gradual upward trend from 30 °C, reaches a temporary peak at the range of 65 °C to 75 °C, and then slightly decreases, under the aging methods of UV radiation and TFOT. In addition, the phase angle aging index also presents a similarity to that under solution aging. However, significant differences are recorded in the aging index under TFOT+UV aging method. The complex modulus aging index mounts up very quickly with the temperature increasing from 30 °C to 80 °C but reaches the highest level hardly. The reason for this behavior is related to the heavy aging degree that makes the temperature barrier rheological property hard to break.

Compared to LFA, the larger complex modulus aging index and smaller phase angle aging index of SSF are presented under all the three aging methods, which is accordant to the trend of temperature sweep analysis. Similarly, the complex modulus aging index under UV aging shows the greatest difference. The analysis results of Figure 10 reveal that the addition of SSF in bitumen performed poorly to resist the aging method of TFOT/UV/TFOT+UV, especially under UV radiation. This phenomenon could be correlated with the porous structure in SSF, which would cause multiple reflections of UV radiation then a heavier aging degree. Additionally, heat and oxygen are also accessed to pores more easily, resulting in a poor resistance to TFOT aging.

#### 4.3.2. Self-Healing Properties

The self-healing properties of the asphalt mastics are measured by frequency sweep and calculated by Equation (3), which represents the relationship between frequency and complex viscosity.

(3) $$\eta^{*}=m{\left|f\right|}^{n-1}$$

In Equation (3), *f* stands for the test frequency, η* stands for complex viscosity, *m* and *n* are the fitting parameters (*n* is also named the flow behavior index). When the parameter n is equal to 1.0, the measured asphalt mastics corresponds to Newtonian fluid, and when the parameter n is less than 1, the measured asphalt mastics shows higher pseudoplasticity. When the flow behavior index is between 0.9 and 1, samples can be regarded as close to Newtonian behavior. In this study, the temperature that corresponds to the flow behavior index equaling 0.9 is determined as the initial self-healing temperature.

**Figure 11:**
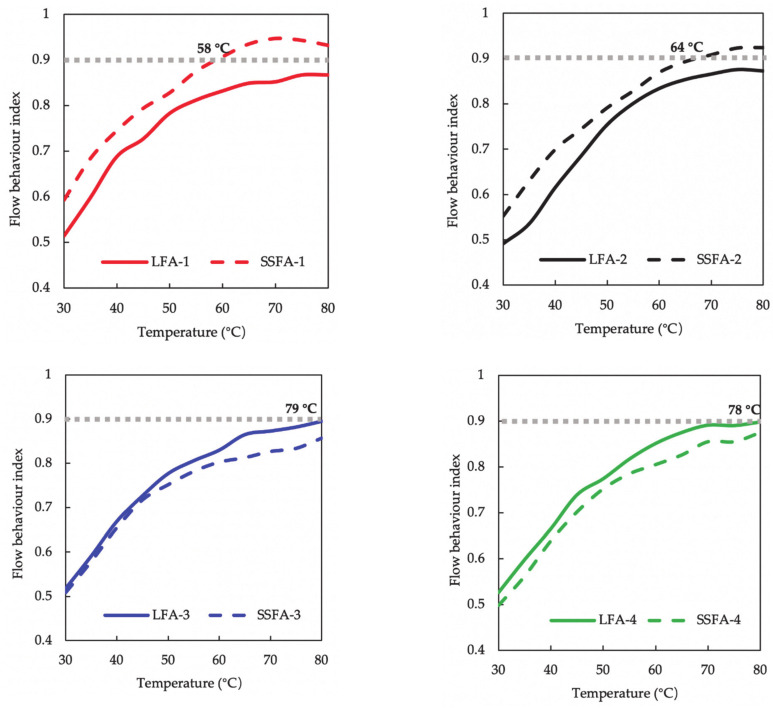

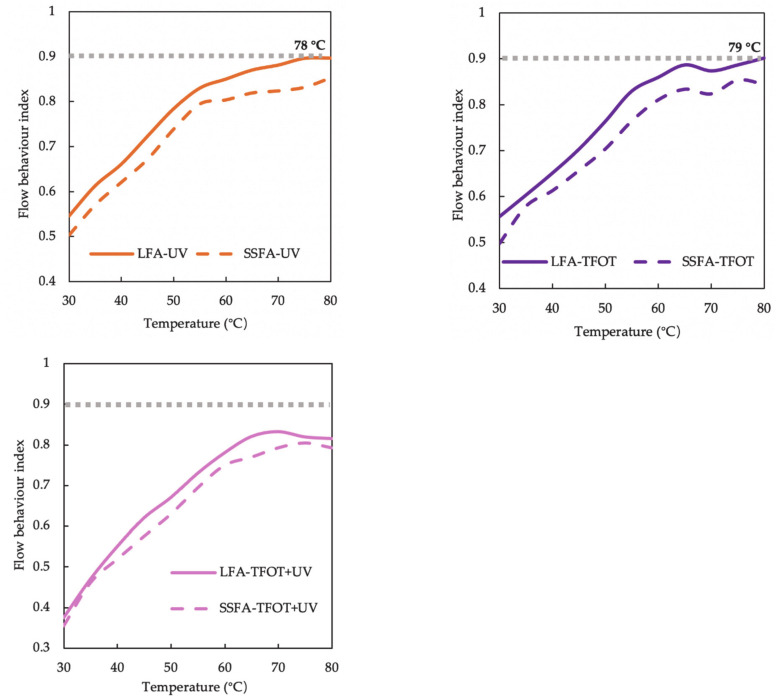
The comparison of SSFA and LFA in self-healing property under various aging methods.

The flow behavior indices of the asphalt mastics under different aging methods are shown in Figure 11. The calculation results show obvious deviations between SSFA and LFA. The flow behavior indices of SSFA are smaller than that of LFA under the aging methods of NaCl solution, pure water, TFOT, UV and TFOT+UV, while opposite under the aging methods of acid solution and alkaline solution. The definite initial self-healing temperatures of SSFA are 58 °C and 64 °C under the aging methods of acid solution and alkaline solution, respectively. As for LFA, the temperature is around 79 °C under the aging methods of NaCl solution, pure water, TFOT and UV. Particularly, SSFA and LFA hardly reach 0.9 due to the fact that the aging degree of TFOT + UV is so heavy.

#### 4.3.3. Chemical Functional Group Analysis

The carbonyl functions C=O (centered around 1700 cm^−1^) are monitored by studying their changes in spectra. The carbonyl functions C=O can offer the information about oxidation of bitumen. The chemical functional group of bitumen in asphalt mastics can be evaluated by the relative contents of the carbonyl group (C=O) defined as Equation (4).

(4) $$I_{C=O}=\frac{A_{1703}}{\sum A}$$

where *A*_1703_ are the areas of the absorption bands at 1703 cm^−1^, ΣA is the sum of the areas of all the absorption bands.

**Figure 12:**
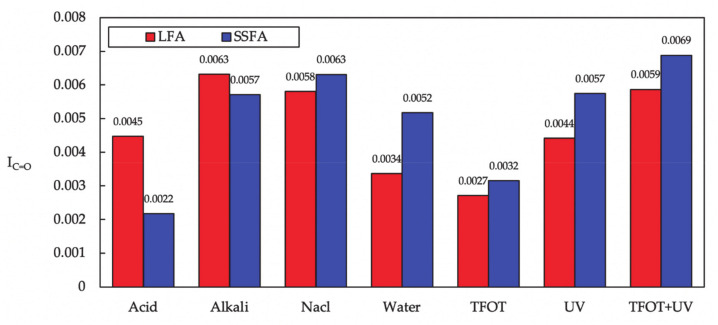
I_C=O_ of bitumen in asphalt mastic under different aging methods.

From Figure 12, the order *I*_C=O_ values of SSFA are NaCl > Alkali > Acid > Water and TFOT+UV > UV >TFOT while the order *I*_C=O_ values of LFA are Alkali > NaCl > Water > Acid and TFOT+UV > UV >TFOT, indicating the hazard effect of these aging methods. In addition, *I*_C=O_ values of SSFA are smaller than that of LFA under alkali solution and acidic solution. Generally, the molecules of bitumen would react with the acid solutions, which can take the hydrogen atoms away and expose the oxygen atoms, and in the alkaline solutions, carboxylic acids in the bitumen can make reaction with alkaline substance. However, the abundant f-CaO in SSF tends to react with the components in the solutions faster than Fisher esterification and the neutralization reaction, preventing the aging to the bitumen.

## 5. Conclusions

(1) In comparison of LF, characterization of SSF indicated similarity but more uniform in particle size distribution, which is related to the preparation methods of the fillers. On the contrary, distinction in surface characterization and pore characterization of the fillers differs significantly. SSF performs rougher surface and more abundant pore characterization, whereas LF has a relatively smooth surface and dull pore characterization.
(2) The addition of SSF and LF to asphalt binder results in an increment in the complex modulus and the properties of rutting resistance and fatigue resistance of asphalt mastic. The enhanced effect of SSF resembles that of LF, confirming the feasibility of replacement which was raised in previous research.
(3) Under the same condition of aging time, SSFA performs better in the environment of acid solution and pure water, worse in the environment of alkali solution and NaCl solution, as opposed to the performance of LFA. The enrichment of f-CaO in SSF effects the orders of reaction with solutions, due to the fact that the f-CaO tends to conduct the processing more easily.
(4) SSFA presents slightly poorer performance under the aging methods of TFOT/UV/TFOT+UV compared to LFA, which is caused by the pore structure in SSF reflecting less energy and refracting more energy more easily. More consumption of bitumen has the potential to improve the impact, and effective measurement to delay the impact needs further investigation in future research.

## Figures and Tables

**Table 1:** Properties of AH-70 asphalt and steel slag.

Materials	Properties	Values	Specifications [28]
AH-70 asphalt	Penetration (25 °C, 0.1 mm)	63.6	60~80
	Ductility (15 °C, cm)	>100	≥40
	Softening point (°C)	47.8	43
	Density (g/cm^3^)	1.035	-
Steel slag	Fine aggregate density (g/cm^3^)	3.56	≥2.9
	Coarse aggregate density (g/cm^3^)	3.65	≥2.9
	Los Angeles abrasion	8.3	≤28
	Crush values	12.9	≤26

**Table 2:** Properties of steel slag fillers and limestone fillers.

Properties		Steel Slag Fillers	Limestone Fillers
Apparent specific gravity (g/cm^3^)		3.640	2.708
Size range	<0.6 mm	100	100
	<0.15 mm	100	99.2
	<0.075 mm	100	94.6

**Table 3:** Pore characteristics of SSF and LF.

Pore Characteristics	Units	SSF	LF
BET surface area	m^2^/g	1.2906	0.6448
Single point adsorption total pore volume of pores	cm^3^/g	0.006174	0.002228
BJH desorption average pore diameter (4 V/A)	Å	180.211	138.726

## Data Availability

The data presented in this study are available on request from the corresponding author.

## List of Acronyms

**Acronyms**	**Full Name**
LF	Limestone filler
SSF	Steel slag filler
VA	Virgin asphalt
LFA	Limestone filler asphalt mastic
SSFA	Steel slag filler asphalt mastic
